# Coaching Through Technology: A Systematic Review into Efficacy and Effectiveness for the Ageing Population

**DOI:** 10.3390/ijerph17165930

**Published:** 2020-08-15

**Authors:** Roberta Bevilacqua, Sara Casaccia, Gabriella Cortellessa, Arlene Astell, Fabrizia Lattanzio, Andrea Corsonello, Paola D’Ascoli, Susy Paolini, Mirko Di Rosa, Lorena Rossi, Elvira Maranesi

**Affiliations:** 1 Scientific Direction, IRCCS INRCA, 60124 Ancona, Italy; r.bevilacqua@inrca.it (R.B.); f.lattanzio@inrca.it (F.L.); p.dascoli@inrca.it (P.D.); l.rossi@inrca.it (L.R.); e.maranesi@inrca.it (E.M.); 2 Department of Industrial Engineering and Mathematical Sciences, Polytechnic University of Marche, 60121 Ancona, Italy; s.casaccia@staff.univpm.it; 3 CNR–Italian National Research Council, ISTC, 00185 Rome, Italy; gabriella.cortellessa@istc.cnr.it; 4 Occupaitonal Sciences & Occupational Therapy, Univeristy of Toronto, Toronto, M5G 2A2 ON, Canada; arlene.astell@utoronto.ca; 5 Unit of Geriatric Pharmacoepidemiology and Biostatistics, IRCCS INRCA, 60124 Ancona, Italy; a.corsonello@inrca.it; 6 Unit of Neurology, IRCCS INRCA, 60124 Ancona, Italy; s.paolini@inrca.it

**Keywords:** coaching, technology-integrated intervention, older people, self-management, Intrinsic Capacity, virtual agent, avatar, robot, health literacy

## Abstract

*Background*: Despite the evidence on the positive role of self-management, the adoption of health coaching strategies for older people is still limited. To address these gaps, recent efforts have been made in the ICT sector in order to develop systems for delivering coaching and overcoming barriers relating to scarcity of resources. The aim of this review is to examine the efficacy of personal health coaching systems for older adults using digital virtual agents. *Methods*: A systematic review of the literature was conducted in December 2019 analyzing manuscripts from four databases over the last 10 years. Nine papers were included. *Results*: Despite the low number of studies, there was evidence that technology-integrated interventions can deliver benefits for health over usual care. However, the review raises important questions about how to maintain benefits and permanence of behavior change produced by short-term interventions. *Conclusion*: These systems offer a potential tool to reduce costs, minimize therapist burden and training, and expand the range of clients who can benefit from them. It is desirable that in the future the number of studies will grow, considering other aspects such as the role of the virtual coaches’ characteristics, social-presence, empathy, usability, and health literacy.

## 1. Introduction

Chronic health conditions and multimorbidity are well-known issues facing our ageing society. According to the recommendations of the Joint Action on Chronic Diseases and Promoting Healthy Ageing across the Life Cycle (JA-CHRODIS) [1], multiple strategies should be implemented to address the complex health needs of the older population. Providing opportunities for improving the self-management capabilities of older adults is described as a key component of any innovative user-centered care pathway tailored to older people with chronic conditions [2,3,4].

There is much evidence, in fact, highlighting the positive role of self-management for both prevention and daily management of chronic illness [5], in terms of optimization of lifestyle [6], increased adherence to treatments, positive health outcomes, and support to patients’ resilience [7], and thus resulting in the improvement of the Intrinsic Capacity (IC), the composite of the individual’s cognitive and physical functions, that represents the target of any multicomponent healthcare interventions integrated with technology [8].

Due to the heterogeneity of the ageing population, characterized by different levels of intrinsic capacity [9], disability [10], and motivation, personalized health coaching may represent an effective way to encourage self-management. Health coaching can be described as a behavioral intervention to promote goals and reduce health risks. One approach to health coaching is through a directive and client-centered counselling technique, the Motivational Interview (MI), a therapeutic approach developed to uncover a client’s motivation to change their behavior [11].

MI-based health coaching has been shown to be effective in improving overall well-being, managing medication and adherence to treatment in case of chronic diseases, and promoting physical activities and appropriate nutritional and lifestyle habits [12,13], including weight loss [14], quality of life in case of chronic disease [15], and physical activity [16]. A recent randomized controlled trial with 250 patients living with T2DM found that the group who received coaching through MI experienced a significant improvement in self-management and quality of life in comparison to the control group [17].

Recently, efforts have been made in different areas of the ICT sector to develop systems to deliver coaching to older people, in order to overcome the issues relating to scarcity of personal and societal resources. A qualitative synthesis of technology-delivered adaptations of MI [18] identified 41 studies covering a broad range of populations, including substance use, risky sexual behavior, and reducing blood pressure, as well as across age ranges from childhood to late life. Interestingly, none of the studies in the review included an RCT, leaving the authors unable to draw conclusions about the efficacy of technology-delivered adaptations of MI.

Since this review, there have been further advances in technology, creating even more opportunities for offering personalized health coaching to older adults. This includes the use of avatars and robots as agents to promote adherence and maintain motivation.

The aim of this review is to examine the efficacy of personal health coaching systems for older adults using digital virtual coaches. For the study, the following definition of virtual coach were adopted: “[…] as computer systems capable of sensing relevant context, determining user intent and providing useful feedback with the aim of improving some aspect of the user’s life” [19]. Following this definition, the most relevant capabilities of an e-coach should be teaching new skills, establishing an effective relationship based on trust, and providing relevant and accurate information to users that can be provided only partially through web/online resources [20].

The findings will also be used to inform future design and developments.

## 2. Materials and Methods

### 2.1. Literature Search and Study Selection

The methodology of this systematic review was based on the Preferred Reporting Items for Systematic Reviews and Meta-Analyses (PRISMA) guidelines with the main aim of mapping all the available studies devoted to evaluate the efficacy of technological systems/digital agents for health coaching, following an evidence-based approach (Randomized Controlled Trials (RCT) study design). A systematic review of the literature was conducted in December 2019. The data were collected from PubMed, Scopus, Embase and Elsevier databases, analyzing manuscripts and articles of the last 10 years (from January 2009 to November 2019), in order to obtain the latest evidence in the field.

Based on consultation with the multidisciplinary research team, health coaching technique studies and applications related to behavioral intervention were searched using the following search terms, and the combinations thereof: old*, coach*, randomized controlled trial, health promotion, self-management, motivation, conversational agent, avatar, virtual, and digital.

After the preliminary search, 994 articles resulted from PubMed, 73 from Scopus, 442 from Embase, and 677 from Elsevier.

The findings were analyzed and screened by four experts of the team: a bioengineer, a clinical neuropsychologist, a statistician, and a geriatrician. In particular, three review authors independently reviewed titles and abstract retrieved from the search in order to determine if they met the predefined inclusion criteria. The full text articles were subsequently analyzed.

The first screening was based on the analysis of the title and deduplication of the findings. After the first step, 99 articles resulted from PubMed, 42 from Embase, 0 from Scopus and Elsevier. A second screening was based on abstract analysis. After this step 18 papers included from Pubmed and 12 from Embase. Another researcher (a statistician) confirmed the accuracy of the papers selection and screened for any possible omission.

### 2.2. Selection Strategy

We included RCTs written in English aimed to study the use of technology for coaching. Thus, we selected studies meeting the following criteria:

1. Studies conducted on adult aged ≥65 years.
2. Studies devoted to use technological systems for health coaching, in multiple or sole interventions, without any restriction in terms of technological applications.
3. Randomized controlled trials, with control group which received usual care or different intervention.
4. Before-after comparison of a single group.

On the contrary, we excluded studies that met the following criteria:

1. Conference proceedings.
2. Studies for which the full text was not found.
3. Studies written in languages other than English.
4. Technical papers.
5. Qualitative studies.
6. Review articles.

### 2.3. Data Collection

**Figure 1:**
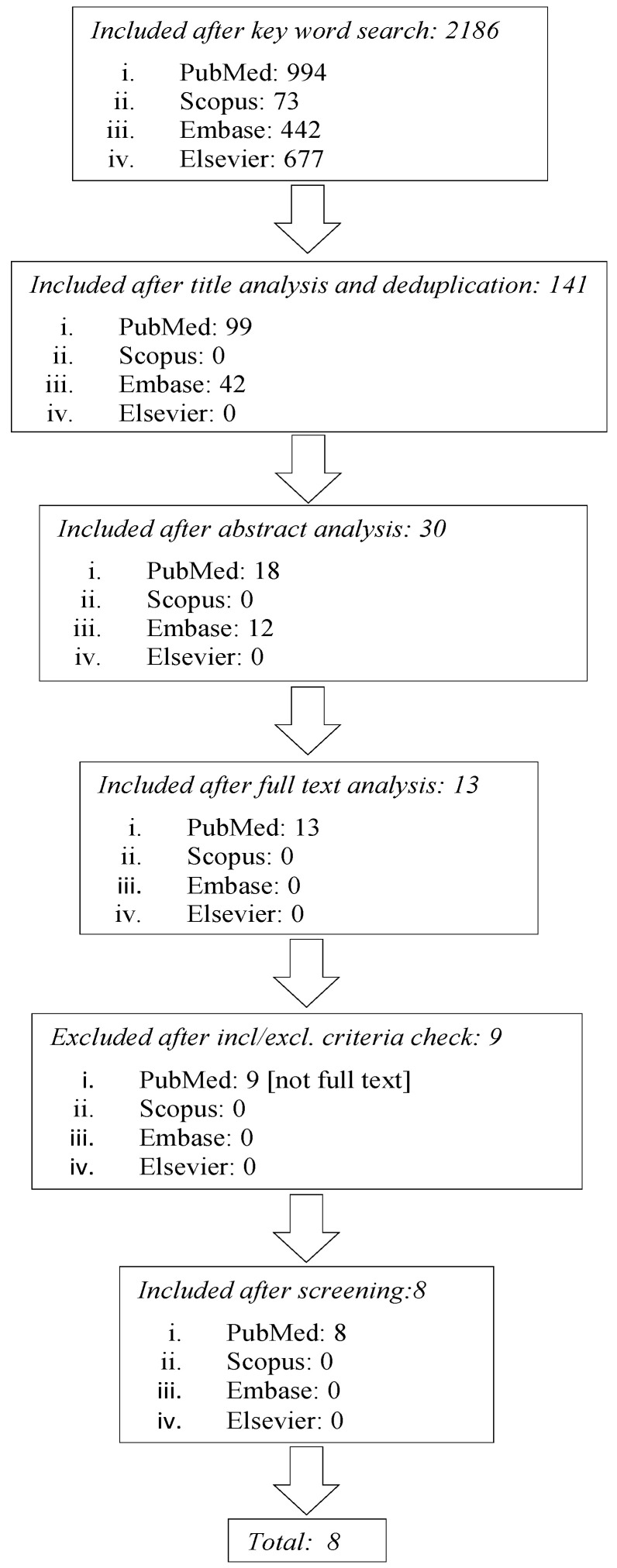
The flowchart search strategy.

After the screening based on the inclusion/exclusion criteria, conducted on the full text articles, the studies were selected as follows: eight from PubMed and zero from Scopus, Embase, and Elsevier database. Figure 1 shows the flowchart search strategy applied.

## 3. Results

A total of eight papers were included [21,22,23,24,25,26,27,28].

### 3.1. Study Quality Evaluation

Quality evaluation was performed based on the PEDro scale, suggested for evidence-based reviews [29]. The final score was settled when the three authors reached agreement after repeated review and analysis. Of the nine studies considered, the PEDro score ranged from 5 to a maximum of 7 (Table 1).

### 3.2. General Characteristics of the Study Population

All the studies were focused on older people with a mean age of 67.06 (±7.9) years for the experimental group and 66.8 (±6.7) years in the control group. The number of participants involved in all the studies is 1182, ranging from 18 to 478. There were 517 were males and 665 females.

The participants considered are divided into two main categories: healthy elderly, not affected by chronic diseases (*n* = 561), and patients with chronic obstructive pulmonary disease (*n* = 562).

### 3.3. Descriptive Analysis and Outcome Measures

Table 2 shows the characteristics of the studies. The outcome could not be pooled into meta-analysis due to the following reasons: clinical heterogeneity can be clearly observed from the participant, intervention, exercise mode, and outcome measures of the included studies.

### 3.4. Intervention Effects

Eight papers report the results of clinical trials involving a group of patients that performed training with virtual coach versus a control group that received usual care training [21,22,23,24,25,26,27,28]. The period of the virtual coaching ranged from 1 to 12 weeks. Moreover, in one paper, a robot was used to carry out the self-management program [26]; in another one conversational agents were used [21]; and in the other six, a virtual coach, who can be an avatar [25], a digital coach who uses messages or emails [27], or an activity coach [22,23,24,28], was used.

Generally, all experimental groups (EGs) in the studies received only the technological coaching, aimed at achieving different goals such as the improvement of physical activity or the enhancement of self-management, while the control groups (CGs) have received only traditional care. The studies reported varying lengths of follow up: 1, 3, 6, 9, or 12 months.

The study of Bickmore et al. [21] involved 263 sedentary older adults randomized and stratified according to clinical state and health literacy status. The intervention group was provided with a tablet to use for two months connected to a pedometer. The patient interacted with the computer-animated virtual exercise coach daily to discuss walking and to set walking goals. Daily 5 min conversations with the embodied conversational agent were designed to promote health behavior change. Control participants were given a pedometer intervention with no coaching. The experimental group walked significantly more steps than the control group at 2 months, but this effect waned by 12 months.

The study by Mc Donald et al. [22] involved 18 older adults with pain from osteoarthritis. Participants were randomly assigned to the virtual pain coach plus pain communication education group, or to the pain communication education-only group. Participants in the virtual pain coach group practiced talking about their osteoarthritis pain with the virtual coach and then they had ambulatory medical visit. Results showed no significant differences between the groups in pain intensity or depression. Many older adults in the virtual pain coach group reported a change from nonuse to use of opioids at 1 month, 50% vs. 0% of the education only group.

In a study by Ritchie et al. [24], 478 people with chronic heart failure (CHF) and chronic obstructive pulmonary disease (COPD) were recruited in order to clarify how technology can best support patients during their transition from the hospital. The virtual coaching program—E-Coach—consisted of an interactive voice response system, with condition-specific customization, a daily dashboard and in-hospital and post-discharge support by a care transition nurse. Results showed that the COPD patients but not the CHF patients who used E-Coach, had significantly fewer days in hospital, but overall, the E-Coach did not reduce rehospitalization.

Another COPD study [26] involved 60 patients, randomized to receive a robot at home for 4 months or to a control group. Results showed no significant differences in the number of respiratory-related hospitalizations or in quality of life between the groups. Nevertheless, the intervention group increased their rehabilitation exercise frequency compared with the control group.

In a study by Tabak et al. [23], 34 patients with COPD were recruited and randomized into two groups: an intervention group that received a telerehabilitation consisting of an activity coach (3D-accelerometer with smartphone) for ambulant activity registration plus real-time feedback and a control group. Results showed that activity level was not significantly affected by the telerehabilitation intervention, but health status significantly improved within the intervention group.

A second study by Tabak et al. [28] investigated the use and satisfaction of clients with COPD with a telehealth program. Twenty-nine patients were randomly assigned to the intervention group (virtual program for 9 months) or to the control group (usual care). The intervention program consisted of an activity coach for ambulant monitoring and real-time coaching of daily activity behavior, web-exercise program for home exercising; self-management of the disease, and teleconsultation. Results showed that patients were satisfied with the received care, and that parts of the program were highly used during the intervention period. Nevertheless, patient adherence with the exercise scheme was low.

The objective of Andrade et al. [25] was to determine whether an avatar-based, online, self-management program is an effective therapeutic approach for women with overactive bladder (OAB). To do this, the authors recruited 41 women with symptoms of OAB for at least 3 months and randomized them into two groups to receive an online self-management program with a generic avatar coach or an identical online program with voice only. Results showed that the group that received the self-management with the avatar coach reported significant improvements in the quality of life.

Broekhuizen et al. [27] studied 235 inactive older adults, to assess the effectiveness of an internet-based intervention on improving quality of life of this population by increasing physical activity. The intervention comprised an internet program aimed at increasing physical activity using monitoring and feedback by accelerometer and feedback by digital coaching (*n* = 119). The control group received no intervention (*n* = 116). After 3 months, a significant improvement in quality of life was seen in the experimental group, particularly in participants who reached their individually targeted increase in daily physical activity.

## 4. Discussion

This review highlights the scarcity of randomized controlled trials of technology-delivered interventions using virtual coaches to deliver health coaching to older adults. There was also a wide range of technology utilized in the studies including wearables, smartphones, avatars, and robots. The duration of interventions also varied from 1–12 weeks, with follow-ups ranging from one to 12 months. The number of studies, often with small numbers of participants, different interventions and durations, all affect the interpretation of their potential impact.

However, from the small number of studies reviewed, there was evidence that technology-integrated interventions may deliver benefits over usual care. For example, an embodied conversational agent increased daily walking in older adults after two months, although this declined over a year. Another important finding is the impact of a virtual coach (E-Coach) on reducing 30 day re-hospitalization rates in patients affected by Chronic Obstructive Pulmonary Disease (COPD), when associated with personal contact with care staff [24]. Moreover, a human-like-avatar based intervention, associated with an online self-management training, has been demonstrated to be more effective in the management of overactive bladder, if compared to the same intervention without the presence of the avatar [25].

The review also raises important questions about how to maintain benefits gained from a fixed-term intervention but also about the stability or permanence of behavior change produced by short-term interventions. This was illustrated by one of the COPD telehealth interventions conducted in the Netherlands by Tabak and colleagues [28], where there was high adherence to the intervention, which was used on 86% of treatment days. However, analysis of the way the four components comprising the intervention—Web-based exercise programme, digital activity coach (accelerometer and smartphone), web-based self-management module and teleconsultations with personal physiotherapist—were used revealed that the self-management module, which was provided by their health professional in an interactive real-life group, was very high but the use of the exercise module and activity coach was extremely low [28].

None of the identified studies used Motivational Interviewing (MI) to inform their contents or delivery style. This is interesting given that MI has been shown to be effective in supporting patients, young and old, to self-manage. However, MI-based digital interventions are starting to emerge for chronic health management [30].

Recently, the promotion of health literacy competences through the virtual coaches is achieving attention, as it is defined as “the degree to which people are able to access, understand, appraise and communicate information to engage with the demands of different health contexts in order to promote and maintain good health across the life-course” [31].

In line with this, our results have shown that virtual coaches are useful means to vehiculate new competences, in particular in enabling self-management to enhance critical health literacy skills [24]. A recent study from Bickmore et al. [32] has shown that the level of health literacy mediates the perception of the virtual coaches by the older people, as the patients with inadequate health literacy seem to prove beneficial from the personification of the virtual coach, resulting in increased adherence to the recommendations and therapeutic alliance.

This is in line also with a recent review in the field [33], as the authors conclude that virtual agents should not simply make a user-friendly coaching intervention, but they should build empathy and feeling of trust in the e-coach, in order to promote a solid change of unhealthy behaviors.

The evidence from the present review and other not randomized trials in this field may suggest a double role of health literacy as mediator of the acceptance of the virtual agents, on one side, and target of the coaching intervention on the other. The next studies in the field should approach the role of the characteristics of the virtual coaches, including level of usability and accessibility in providing adequate support to the older people, in terms of impact on health outcomes, in order to evaluate the effectiveness of technological coaching interventions also on a non-technologically literate population.

Even if virtual agents, such as avatars and robots, seem to be able to support the provision of effective coaching strategies, there is no available evidence that can clarify which are the characteristics of such technologies that can contribute the most in improving selected health outcomes, adopting an evidence-based approach. From the literature, it is well-known that a link exists between the social presence dimension of the virtual agents and the level of trust by the users [34,35]. Due to unavailability of evidence from RCT studies, it is possible only to assume that the more the virtual agent is able to provide empathy and social presence, the more the users will be inclined or persuaded to trust and follow the coaching advice, but the direct impact on health of advanced interactive competences of virtual coaches must still be proved in further studies.

In terms of new developments, these include coaching systems deploying sensors and devices to measure user behaviors related to health management, emotional status, and providing advice. An important part of the overall technology is characterized by Artificial Intelligence and Machine Learning solutions [36,37]. Tailored coaching approach can be applied on the user through the use of AI algorithms on the analyzed data coming from the older users and the home environment. In particular, an accurate measurement process together with sophisticated data analysis methodologies [38], e.g., machine learning, can provide elaborated information focused on the user’s behavior that can be used for a more appropriate coaching approach. Starting from the first generation of ICT solutions, designed basically to remind the intended users to do useful activities, these systems have advanced to incorporate the interaction capabilities of virtual agents, such as avatars and social robots [39,40,41]. For example, Fiorini, et al. [42] developed a service model comprising a hybrid robot-cloud approach to self-management of chronic disease. Further evidence about their effectiveness and efficacy is required, by adopting appropriate study design, as Randomized Controlled Trial and innovative theoretical framework, like the WHO’s Intrinsic Capacity, in order to understand the impact of virtual coaches on health and self-management capabilities in the older people.

Despite the wealth of the review, the present article presents some limitations. First of all, quasi-experimental studies, technical, and design papers were excluded from the dissertation. Even if our focus was to understand the efficacy of innovative coaching services integrated with technology, nonetheless it is important to consider all the constraints related to this research area, first of all the difficulty in conducted experimental studies with a large number of users, due to the cost of development of technologies, for example. In turn, this may explain the low number of studies retrieved in form of RCTs, which may lead to a complete understanding of the efficacy of coaching intervention through digital agents, in an evidence-based perspective.

Another limitation is related to the terms used by the scientific community, including “self-management”, “self-care”, “coach”, and “virtual or digital agent”, to define health interventions that promote the primary role of the older person and the availability of a technological mentor to achieve positive health outcomes.

## 5. Conclusions

The aim of this review was to investigate the studies on the effectiveness of ICT-based coaching systems in providing support in the management of the health of older people, focusing on the Randomized Clinical Trial as investigation target. The review was based on the PRISMA methodology and led to the selection of eight RCT works.

A complete revision of the terms in the field should be conducted by a multidisciplinary panel of experts, in order to reach a consensus on definitions and thus simplify the research in the field, providing evidence through an innovative methodological approach.

Although the number of ICT-based coaching systems is relatively high, there are still few RCT studies to be able to convincingly demonstrate their effectiveness. Nevertheless, some interesting results emerged: in one case there was a direct association between the use of a virtual trainer and the increase in physical activity; in addition, the coaching systems integrated in technology have shown positive effects on health outcomes even in the case of complex chronic diseases. Some of the results have shown that virtual coaches are useful means of conveying new skills, in particular in allowing self-management to improve health literacy skills.

Altogether these systems offer a potential tool to reduce costs, minimize the burden of the therapist and training and expand the range of clients who can benefit from them. It is desirable that in the future the number of studies will grow, considering other aspects, such as the role of the characteristics of virtual coaches, social presence, empathy, and also the level of usability, in providing adequate support for the health of older people [43].

## Figures and Tables

**Table 1:** Scores of methodological quality assessment of the included studies.

PEDro	Bickmore et al., 2013 [21]	Mc Donald et al., 2012 [22]	Tabak et al., 2014 [23]	Ritchie et al., 2012 [24]	Andrade et al., 2014 [25]	Broadbent et al., 2018 [26]	Broekhuizen et al., 2016 [27]	Tabak et al., 2013 [28]
Eligibility	Y	Y	Y	Y	Y	Y	Y	Y
Randomized Allocation	Y	Y	Y	Y	Y	Y	Y	Y
Concealed Allocation	Y	N	Y	Y	Y	Y	Y	Y
Baseline Comparability	Y	N	N	N	N	N	N	N
Blinded Subject	N	N	N	N	N	N	N	N
Blinded Therapists	N	N	N	N	N	N	N	N
Blinded Raters	Y	N	N	N	Y	N	N	N
Key Outcomes	Y	Y	Y	Y	Y	Y	Y	Y
Intention to Treat	N	N	N	N	N	N	N	N
Comparison between Groups	Y	Y	Y	Y	Y	Y	Y	Y
Precision and Variability	Y	Y	Y	Y	Y	Y	Y	Y
	7/11	5/11	6/11	6/11	7/11	6/11	6/11	6/11

Y: yes. N: no.

**Table 2:** Descriptive analysis of the included clinical studies.

	Population	Intervention	Outcomes and Measurements	Results
Bickmore et al., 2013 [21]	EG = 132 cases (age 71.7 ± 5.6 years) CG = 131 controls (age 70.8 ± 5.2 years)	EG was provided with touch screens tablet to use for 2 months connected with pedometers, that communicate through an animated virtual coach to discuss walking and setting goals.	Primary outcome: Average daily steps for 30 days before the 12-month interview Secondary outcome: Average daily steps for 30 days before the 2-month interview Outcomes were stratified according to health literacy level.	Primary outcome: *p* = 0.09 *p* = 0.02 (with adequate health literacy) Secondary outcome: *p* = 0.01 *p* = 0.03 (with adequate health literacy)
Mc Donald et al., 2012 [22]	EG = 8 cases with osteoarthritis pain (age 69.9 ± 7.4 years) CG = 10 controls with osteoarthritis pain (age 66.7 ± 4.3)	All participants viewed the pain communication videotape. EG practiced talking about osteoarthritis pain with the virtual pain coach	Primary outcomes: Pain intensity Pain interference with activities Depressive symptoms At baseline and 1 month later, between and within groups	Primary outcomes: *p* = 0.72 (between groups) *p* = 0.017 (within EG in depressive symptoms)
Tabak et al., 2014 [23]	EG = 12 cases with COPD (age 64.1 ± 9.0 years) CG = 12 controls with COPD (age 62.8 ± 7.4 years)	4 modules: 1. Activity coach for ambulant activity monitoring and real-time coaching of daily activity behavior; 2. Web-based exercise program for home exercising; 3. Self-management of COPD exacerbations; 4. Teleconsultation.	Primary outcomes: Client Satisfaction Questionnaire 8	Primary outcomes: Satisfaction with received care was 26.4 for the telehealth group and 30.4 (1.5) for the usual-care group after 1 month. After 3 months, this was 26.3 for the telehealth group and 29.9 for the usual-care group.
Ritchie et al., 2012 [24]	EG = 233 cases with CHF/COPD (age 63.0 ± 12.1 years) CG = 245 controls with CHF/COPD (age 63.8 ± 12.8 years)	E-Coach: intervention with condition-specific customization and in-hospital and post-discharge support by a CTN, interactive voice response post-discharge calls, and CTN follow-up versus usual post-discharge care.	Primary outcome: 30-days rehospitalization Secondary outcomes: Rehospitalization/death Community tenure	Primary outcome HR (95%CI): CHF: 1.14 (0.67 1.96) COPD: 0.56 (0.23 1.38) Secondary outcome: Rehospitalization/death: HR (95%CI) CHF: 1.03 (0.6 −1.8) COPD: 0.44 (0.2 −1.2) Community tenure: Beta(95%CI) CHF: −0.11 (−1.0 −0.9) COPD: 1.12 (1.11 −2.12)
Andrade et al., 2014 [25]	EG = 22 cases with OAB (age 62.41 ± 7.25 years) CG = 19 controls with OAB (age 60.68 ± 5.30 years)	EG: self-management program with a generic avatar coach with a self-avatar peer mentor. CG: identical online program with voice only.	Primary outcome: Quality of life (HRQoL) Secondary outcomes: Perception of bladder condition (PPBC) OAB symptoms Self-efficacy	Primary outcome: HRQoL *p* = 0.02 Secondary outcomes: PPBC *p* = 0.63 OAB symptoms *p* = 0.75 Self-efficacy *p* = 0.99
Broadbent et al., 2018 [26]	EG = 30 cases with COPD (age 69.10 ± 9.85) CG = 30 controls with COPD (age 70.57 ± 10.34)	EG received a robot at home for 4 months in addition to usual care. CG received standard care alone.	Primary outcome: Number of days of hospitalization. Secondary outcome: Medication adherence Frequency of rehabilitation exercise Quality of life using CCQ	Primary outcome: *p* = 0.9 Secondary outcomes: Medication adherence: *p* = 0.04 Frequency of rehabilitation exercise: *p* = 0.001 CCQ: *p* = 0.35
Broekhuizen et al., 2016 [27]	EG = 119 cases (age 64.7 ± 3.0) CG = 116 controls (age 64.9 ± 2.8)	EG used internet program with monitoring and feedback by accelerometry and feedback by digital coaching (messages, e-mail). CG received no intervention.	Primary outcomes: Quality of life (RAND-36) Physical activity between and within groups	Primary outcomes: Between groups RAND-36: *p* = 0.03 Physical activity: *p* = 0.01 Primary outcomes: Within EG RAND-36: *p* = 0.009 Physical activity: *p* = 0.004
Tabak et al., 2013 [28]	EG = 14 cases (age 65.2 ± 9.0) CG = 11 controls (age 67.9 ± 5.7)	EG: activity coach for ambulant activity registration and real-time feedback for 4 weeks. CG: usual care	Primary outcomes: Activity level (step/day) Health status (CCQ) between and within groups	Primary outcomes: Between groups Activity level: *p* = 0.48 CCQ: *p* = 0.1 Primary outcomes: Within EG Activity level: *p* = 0.38 CCQ: *p* = 0.03

EG: experimental group; CG: control group; COPD: chronic obstructive pulmonary disease; 6MWT: 6-min walking test; CCQ: clinical COPD questionnaire; MFI: multidimensional fatigue inventory; EQ-5D: EuroQol; BPAQ: Baecke Physical Activity Questionnaire; IMA: integrated modulus of body acceleration; CHF/COPD: heart failure and chronic obstructive pulmonary disease; CTN: care transition nurse; OAB: Overactive bladder; HRQoL: health-related quality of life; CCQ: Clinical COPD questionnaire; TOFHLA: Test of Functional Health Literacy in Adults; RAND-36: Research and development 36-item health survey.
